# Study of Physical Fitness, Bone Quality, and Mediterranean Diet Adherence in Professional Female Beach Handball Players: Cross-Sectional Study

**DOI:** 10.3390/nu13061911

**Published:** 2021-06-02

**Authors:** Alejandro Martínez-Rodríguez, Javier Sánchez-Sánchez, María Martínez-Olcina, Manuel Vicente-Martínez, Laura Miralles-Amorós, Juan Antonio Sánchez-Sáez

**Affiliations:** 1 Department of Analytical Chemistry, Nutrition and Food Science, Faculty of Sciences, Alicante University, 03690 Alicante, Spain; maria.martinezolcina@ua.es (M.M.-O.); lma52@alu.ua.es (L.M.-A.); 2 Alicante Institute for Health and Biomedical Research (ISABIAL Foundation), 03010 Alicante, Spain; 3 Department of Sport Sciences, School of Sport Sciences, Universidad Europea de Madrid, 28670 Madrid, Spain; 4 Faculty of Health Science, Miguel de Cervantes European University, 47012 Valladolid, Spain; mvmartinez11006@alumnos.uemc.es; 5 GDOT Research Group, Faculty of Sport, Universidad Católica de Murcia, 30107 Murcia, Spain; jasanchez419@ucam.edu

**Keywords:** women’s health, bone mineral density, diet, beach sports, osteoporosis

## Abstract

(1) Background: Beach handball is a relatively new type of sport, derived from team handball. The purpose of the study was to evaluate the physical fitness of elite players of this sport by studying some variables of sports performance, including strength, endurance and power, and dietary habits, and to assess bone ultrasonographic variables. (2) Methods: 33 beach handball players have participated in this research; 18 juniors (age: 16.7 ± 0.50) and 15 seniors (age: 24.8 ± 4.71). The athletes’ strength was evaluated using the Handgrip Test on the dominant hand, the height of jump was evaluated by a counter-jump on a contact platform, and velocity, agility, and resistance by the Yo-Yo test. The broadband ultrasound attenuation (BUA) and the sound of speed (SOS) through the calcaneus were also measured. The Mediterranean diet adherence (KIDMED) was the questionnaire used to evaluate eating habits. In the statistical analysis, descriptions and correlations were made between the study variables. (3) Results: Both in the case of the dynamometric hand strength test (*p* < 0.05) and in the lower extremity power test (*p* < 0.01), senior players presented significantly higher values compared to junior players (35.1 ± 3.84 vs. 31.8 ± 3.37 and 35.1 ± 6.89 vs. 28.5 ± 5.69 with the dynamometry and Abalakov tests, respectively). However, no differences were observed in the variables by playing position. Significant correlations between different variables have been established, highlighting negative correlations between BMI and weight with the Abalakov Jump Test and positive correlations between Yo-Yo and BUA, and, between BMI and BUA. (4) Conclusions: Older and trained players are in better physical fitness; high weight and BMI have a negative influence on power, agility, speed, and endurance. In general, adherence to the Mediterranean diet is moderate and it seems evident that there is a beneficial influence of beach handball on bone condition, as measured by ultrasound. However future research should be carried on, including dual-energy x-ray absorptiometry assessments and food intake registers for a whole week.

## 1. Introduction

Beach handball (BH) is relatively a new sport specialty. It has led to arousing the interest of the scientific community in the sport, analyzing the different specific performance variables of BH players (e.g., physical and physiological demands or body composition and anthropometric profile) [1,2,3,4,5,6,7] resulting from the particularities of this discipline (e.g., in-flight throw, spin shot, shoot-out, attack in numerical superiority, or defense in numerical inferiority) [8] and caused by the attack–defense actions of the game cycle [9]. Moreover, the competition format of a BH tournament —two to three games per day for two to three days—makes it necessary to design specific training exercises that respond to the actual physiological needs required by the players [7]. 

In relation to the physiological demands (internal load), BH, like other team sports (e.g., ice hockey or soccer [10,11]), requires intermittent efforts [7]. In this sense, this sport discipline is considered to be high-intensity because most of the playing time, the player is over 80% of the HRMAX [2,4,7,12]. Other characteristics to consider because they affect the internal load of the athlete would be the kinematic variables: (a) distance: 445.6 ± 156.3 to 669 ± 155 m per set, and (b) speed: 4.2 ± 0.6 to 5.7 ± 1.2 m/s [4,7,12].

There are different validated instruments and tests to determine performance variables in team sports, and they can provide the necessary data to assess the physical and physiological profile of the athlete. One of these tests is the Yo-Yo Intermittent Recovery Test Level 1 [13], widely applied in recent decades by researchers in indoor handball [14,15,16,17,18,19,20]. Another of the tests, the use of which is widespread, is the Abalakov Jump Test, used to determine the lower body power and fatigue of athletes [21,22,23,24]. In this case, we used the research of Sánchez-Sáez et al. [7] as a reference study of BH, which determined that there were no significant decreases in jumping ability during the tournament in female beach handball players (*p* > 0.05). 

Regarding muscle strength, the Handgrip Test is used to evaluate the musculoskeletal fitness of the upper extremities [3] and muscle fatigue [7] in BH players. This variable could be considered of vital importance when establishing the performance profile of the BH player since the grip of the ball should be propitiated with sufficient strength both in the control and mastery of the ball, and at the time of execution of the passes or throws. To perform all the actions above, the implementation of an adequate high-performance training program is essential, which includes good eating habits, such as the ones offered by the Mediterranean diet (MD) [25]. In this way, in addition to improving physical capacities, the risk of suffering sports injuries, such as bone fractures, will be reduced [26]. 

Regarding bone quality, it has been observed that in addition to being determined by body composition, genetic/ethnic factors, and hormonal status, it is also related to lifestyle behaviors, such as diet and physical activity, which influence bone gain during growth and bone loss later in life [27]. From a mechanical point of view, changes in bone mineral density (BMD) can be made by inducing mechanical stimuli with loading forces or external loads during skeletal muscle contractions. For this reason, athletes practicing sports, such as beach handball, that require high muscular forces (strength training) or general high impacts (sprinting and jumping), are regularly recommended to work on the improvement of bone mass and density [28]. It appears that exercise activities that combine the tension produced by intense muscle contractions and the mechanical stimulus of ground reaction forces are considered better for bone stimulation [29,30,31] than sports such as swimming, water polo, or rowing, which do not show measurable osteogenic benefits [32]. 

In fact, it has been shown that an optimal nutritional status can enhance the effects of training, speeding up recovery and optimizing body composition [33]. The dietary culture of the Spanish population is within the framework of the Mediterranean diet [34,35,36]. This one is characterized by being a balanced diet that provides sufficient calories in the right proportions through high consumption of vegetables, legumes, fruits, nuts, cereals, and olive oil, moderate consumption of fish, eggs, and dairy products, preferably yogurt or cheese, and a lower intake of meat and less consumption of animal fats [37]. 

Therefore, the main objective of this research was to evaluate the physical fitness of elite female BH players through the study of the determinant variables of sports performance, including strength, endurance and power, and their dietary habits, and to assess bone ultrasonographic variables. This research provides relevant information for coaches and physical trainers when planning the season and training sessions. 

## 2. Materials and Methods

**Figure 1:**
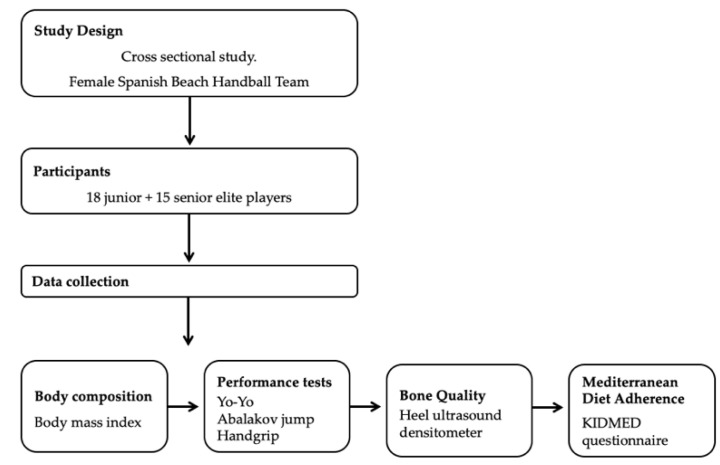
Material and methods scheme.

Figure 1 presents a brief scheme of this section. 

### 2.1. Study Design 

A descriptive, cross-sectional study was conducted to analyze the influence of age category and playing position on the results of performance tests, adherence to the Mediterranean diet, and bone mineral density in female BH players. The research involved the best international players from Spain of this sport modality, who represent elite BH players from all over the world. The Declaration of Helsinki guidelines (revised in Hong Kong in September 1989 and in Edinburgh in 2000) and the recommendations of Good Clinical Practice of the EEC (Document 111/3976/88 of July 1990) were followed for all the procedures carried out. Approval of the research was granted by the Human Research Ethics Committee of the University of Alicante (Spain) (UA-2019-04-09).

### 2.2. Participants and Eligibility Criteria

A total of 33 female beach handball players participated in the study; 54% were juniors and 45% were seniors. For the distribution of the sample by playing position, 6 goalkeepers, 10 wings, 8 specialists, 6 pivots, and 3 defenders participated. All the players who were part of the current Spanish BH national team participated. The following exclusion criteria were considered: the presence of chronic diseases, injury during the training camp preventing the performance of any test, and non-compliance with the informed consent. However, no player refused to participate, and no athlete was excluded; every athlete gave written informed consent. No financial compensation was given to the participants for their collaboration. For those who were minors, consent was given by their parents or legal guardians. The players’ anonymity was always preserved.

### 2.3. Data Collection 

#### 2.3.1. Body Composition

Anthropometric measurements included standing body height (stadiometer accuracy of 0.1 cm; Holtain, Crosswell, Crymych, Pembs, UK) and body mass (0.1 kg; Tanita BF683W scale, Munich, Germany). With the weight and height data, the body mass index BMI (kg/m^2^) was calculated for each of the players.

#### 2.3.2. Performance Tests

The test known as Yo-Yo IR1 was performed in accordance with the procedures described by Krustrup et al. [38]. The test consisted of 20 m runs performed at increasing speeds with 10 s of recovery between runs until the individual became exhausted. Previously, the players performed a warm-up of 5 min of low intensity running, followed by a 5 min warm-up of running at medium-high intensity. The test was terminated when the participant did not reach the first line in time (objective evaluation) twice in a row, or if she felt that she would not be able to complete another sprint (subjective evaluation). The total distance covered was recorded as the “score” of the test. The reliability of the Yo-Yo IR1 is demonstrated by a coefficient of variation (CV) of 3.6% and an ICC of 0.94% [38]. 

The Abalakov Jump Test was used; participants performed 3 countermovement’s with 30 s rests between jumps [39] performed on a stable surface. All the players had to stand upright and perform a 90° knee flexion followed by the fastest possible extension, with the goal of reaching the highest achievable jump height. An optical (infrared) data collection system (Optojump Next Microgate, Bolzano, Italy) was used to calculate the Abalakov jump height. Of the 3 results, the best one was used for statistical analysis. 

A hand-held dynamometer (Takei, Tokyo, Japan) was used with the arm at right angles and the elbows at the sides of the body. The instrument was adjusted, its base rested on the first metacarpal and the handle rested on the middle of the participant’s four fingers. All players performed the test with their dominant hand. Their maximal isometric effort was maintained for 5 s. The test was performed twice, with a 1 min rest between trials, and the highest value being the one to be used in the subsequent analysis.

#### 2.3.3. Bone Quality—Ultrasound Measurement

The bilateral calcanei of each subject were measured using a heel ultrasound densitometer (Achilles EXP II, GE Healthcare, Chicago, IL, USA). To assess bone health, quantitative ultrasound (QUS) measurements are widely used since they are noninvasive measurements, and are also less expensive and simpler than laboratory techniques (e.g., dual-energy X-ray absorptiometry—DXA). Quality assurance was performed using a specific dummy before the first measurement. Along with a coupling medium to ensure good contact, ultrasound gel was applied. During each ultrasonographic assessment, the broadband ultrasound attenuation (BUA) and the speed of sound (SOS) were directly measured. The BUA and SOS measurements, and their derived parameters (rigidity), are seriously associated with fracture risk, while bone quality is the determinant of both bone strength and bone fragility. Therefore, these variables can be used to refer to bone quality [40,41]. The calcaneus stiffness index was calculated as follows [28]: Calcaneus stiffness (A.U.) = (0.67 · BUA + 0.28 · SOS) − 420

#### 2.3.4. Mediterranean Diet Adherence (KIDMED Questionnaire)

The KIDMED questionnaire was published in 2004 to evaluate adherence to the Mediterranean diet (MD) in children and adolescents [42]. The questionnaire constitutes 16 questions, 12 of which represent a positive score in relation to the adherence to the MD, and the other 4 represent a negative score. Positive answers to questions involving greater adherence to the diet are worth +1 point. Positive answers to questions involving less dietary adherence are worth −1 point [43]. Depending on the scores obtained in the questionnaire, the population was divided into 2 groups: (1) poor or average adherence, and (2) excellent adherence.

### 2.4. Statistical Analysis

The Jamovi statistical program (version 1.6.15, Sydney, Australia) was used for data analysis. First, the descriptive data (mean and standard deviation) were calculated. To assess the normality of the descriptive statistics (mean ± standard deviation) and inferential analysis, the Kolmogorov–Smirnov test was performed. To determine homogeneity, the Levene test was used to evaluate the normality of the data (*p* > 0.05). To calculate the differences between the age groups (junior vs. senior), as well as for the analysis of the different variables according to playing position, analysis of covariance (ANCOVA) with Bonferroni correction was used, controlling for the effect of BMI. The effect size was also calculated using a partial eta-squared (ηp2) considering <0.25, 0.26–0.63, and >0.63 as small, medium, and large effect sizes, respectively [44]. 

Correlations between the performance variables (Abalakov, Handgrip, Yo-Yo), the KIDMED total score, bone variables, weight, and BMI (kg/m^2^) were determined utilizing Pearson’s product-moment correlation coefficient (r), with 95% confidence intervals (CI). 

## 3. Results

A total of 33 female BH players (18 junior and 15 senior) of the Spanish Nationality Team participated in this study. Table 1 shows the basic anthropometric and demographic characteristics of the sample. The mean height and body mass were 167 ± 4.90 cm and 62.4 ± 7.29 kg for the junior females, and 169 ± 5.31 cm and 64.9 ± 7.87 kg for the senior players. There were only significant differences in the variable of age (*p* < 0.001), being obviously greater in the senior players. In the rest of the variables, there were no significant differences between the junior and senior players. However, as expected, the senior players showed higher values for weight and height.

The variables of adherence to the Mediterranean diet, sports performance, and bone mineral density are presented in Table 2. Except for the KIDMED total score, the senior players had higher scores for all the variables. In addition, the senior female players present significantly higher values in the case of the dynamometric hand strength test (Handgrip; *p* < 0.05) and the lower limb power test (Abalakov; *p* < 0.01).

The results in Table 3 also indicate differences by position for the BMI, KIDMED total score, performance test, and BDM variables. Post hoc analysis revealed that there were no differences in any of the variables studied because of the playing position.

The correlations between the different variables, separated by age category (junior vs. senior) are presented in Table 4. In the case of junior players, statistically significant negative correlations were observed between BMI and the Abalakov test results (*p* < 0.05), and between weight and the Abalakov (*p* < 0.05). However, significant positive correlations were also observed between the Yo-Yo and Abalakov test results (*p* < 0.05) and between the Yo-Yo test results and SOS values. For the senior players, statistically significant positive relationships were observed between BMI and BUA (*p* < 0.05) and between KIDMED and BMI (*p* < 0.05). However, between the results of the KIDMED and Abalakov (*p* < 0.05), and between BMI and the Abalakov test (*p* < 0.05), the correlations were negative.

## 4. Discussion

The main purpose of this investigation was to assess the physical fitness (through different physical tests), bone mass quality, and adherence to the Mediterranean diet of top-level female beach handball players. The present manuscript studied the differences by age group and playing position, and the correlations between the included variables.

Regarding physical fitness, the senior players were generally heavier and taller than the junior players. Moreover, the seniors scored higher in both the Abalakov jump and Handrip tests. Therefore, the senior players had better lower body power than the junior players (*p* < 0.001). The same occurred with the variable of musculoskeletal fitness of the upper extremities, measured by Handgrip since the senior players had shown higher values (*p* = 0.015). This coincides with previous research conducted with other samples of handball players [25,45]. It seems that the reasons, as demonstrated in female volleyball players [46], are that these differences may be directly related to neuromechanical adaptations because of the repeated actions taking place in training and matches. However, it has also been shown that the results of these tests improved, and therefore, improving physical abilities (jumping, reaction speed, and running speed), when a Plyometric Training Program training was performed [21,22]. This data is of special interest for all the coaches.

In the Yo-Yo Test, there were no significant differences between the values obtained for the junior vs. senior players. One of the reasons why this test was used was because it has been shown [47] that the Yo-Yo Test contributes specifically to the handball performance of elite players, which means that this test, among others, measures important qualities for success in handball. If the results achieved in the present study are compared to those of [20,47], much lower values are observed; the main reason for this distinction is the surface since, in this study, the players did the tests on sand. 

Furthermore, some researchers underline [47] the importance of aerobic and anaerobic qualities for success in handball, supporting the emphasis on the development of prolonged intermittent running ability, sprinting qualities, and repeated sprinting ability in elite handball players. 

In addition, for correlations, negative correlations were observed in the junior players between BMI and the Abalakov jump and between weight and the Abalakov jump. This means that lower weight and lower BMI are related to higher jump heights and therefore, more power in the lower limbs. In previous studies [48] conducted on female handball players, these physical characteristics were already correlated. As already corroborated [49], it seems that an excess of body mass has a negative role in jumping. For the same reason, the senior female players also have negative correlations between BMI and the Abalakov jump. However, significant positive correlations have been observed in the junior players between the results of the Yo-Yo and Abalakov jump tests, and therefore, those players who present greater power in the upper limbs also present greater endurance, agility, and speed.

In terms of bone health, previous studies [50] have demonstrated an osteogenic effect of high-impact exercise (such as BH) and weight training on BMD by DXA. Additionally, a study using DXA and QUS measurements by Lehtonen-Veromaa et al. [51] showed that both femoral neck BMD and heel QUS parameters increased in the following order: control, runners, and gymnasts. In many of the studies performed, in which they have compared an athlete population and a control group, such as the case of female runners [28] or powerlifters [52], it has been observed that athletes who perform a specific sport modality in which there are both impact movements and strength exercises, the values of SOS, BUA, and Stiffness were higher than the control group. Moreover, if the values obtained in the present study of SOS, BUA, and Stiffness are compared for both the junior and senior players, the values of all three parameters are higher than those of female runners [51,53], both long and short distance [28], gymnasts [51], and powerlifters [52]. Other studies [54] also relate higher values to a higher number of hours devoted to training. Therefore, it seems that the fact that beach handball is played on a surface such as sand, and that there are repeated impacts after jumps, turns, and sprints is favored by the development and bone quality of the growing skeleton. 

It should be noted that this is the first study to research the correlation between bone variables (BUA, SOS, and Stiffness) and performance results using different tests that measure strength, endurance, and power. Positive relationships have been observed between SOS values and Yo-Yo test results in the junior players; those players who have managed to run a longer distance (m) are those who have higher values for the speed of sound. These results indicate that athletes’ training endurance, speed, and agility may exert progressive adaptation on the calcaneal bone, tending to produce superior elastic bone strength (this correlates with mineral and protein contents). Furthermore, in the case of the senior players, a positive correlation can be observed between the BMI and BUA values; therefore, those players who have a higher BMI have higher values for broadband ultrasound attenuation. One of the possible reasons could be that players who are taller and heavier have higher bone mineral density, and therefore, a lower risk of fractures.

Regarding the nutrition of the players, positive correlations have been established in senior players between KIDMED and BMI, and thus, those players with higher scores have higher BMI values. Negative correlations have also been found in the senior players between the KIDMED score and the Abalakov jump results; the better the KIDMED score, the lower the Abalakov jump test results, and therefore, lower power. It should be noted that the KIDMED measures the quality of the diet in terms of adherence to the Mediterranean diet; however, it is not able to measure the amount of food eaten by the players. As previously observed [25], adherence to the Mediterranean diet in female beach handball players is moderate. 

It has been previously observed that both correct hydration and nutrition are essential for better performance in beach handball [55,56]. Because this sport is performed in hot environments, female athletes are at greater risk of dehydration and hyperthermia, hindering overall physiological function and cognitive and athletic performance. In addition, sports performance and recovery from exercise have been found to be enhanced by optimal nutrition. Appropriate food choices should be made, in addition to considering sufficient energy intake during both high intensity and long duration training periods, with the goal of maintaining body weight and health and maximizing the effects of training [33]. There is an evident need for athletes to improve their dietary habits, so the intervention of dieticians/nutritionists in multidisciplinary teams is necessary and essential. 

This research has some limitations. Firstly, body composition variables were measured by anthropometry and bone quality by ultrasound. Although both methods are related to bone densitometry (dual-energy X-ray absorptiometry), it was not possible to use this method, which is considered the “gold standard”. In addition, as previously indicated, the quality of the food was evaluated, but it was not possible to assess the quantity. It would be interesting to be able to make 7-day records with a photographic report, to be able to assess both quality and quantity. Due to the fact that there are not many sports played on sand, there are tests, such as the Yo-Yo, that are not validated for this surface, which is why the players presented lower values. Therefore, it would be interesting to validate this test on a sand surface. The Abalakov jump test had to be performed on a regular surface to be able to use the contact platform correctly because the sand was very unstable.

Future research will consider the mentioned limitations. Researchers in the field are invited to provide more specific information regarding the evaluation of physical fitness, body composition, and dietary habits of professional beach handball players, as research is still scarce, as well as regarding the validation of specific tests for this sport on its usual playing surface, sand.

## 5. Conclusions

The results of this cross-sectional study in female athletes from the Spanish BH indicate that older players performed better in the Handgrip and Abalakov jump tests. Correlation analysis highlighted the relationship of weight and poor nutrition with worse results in jumping, agility, power, and endurance tests. Regarding bone mass, it was observed that female handball players have higher values of SOS, BUA, and Stiffness than athletes of other sports disciplines, confirming that sports involving impact, jumps, and sprints have a higher level of bone mineral density.

## Figures and Tables

**Table 1:** Demographic and basic anthropometric characteristics of professional female beach handball players.

Demographic and Anthropometric Variables	Junior (n = 18)			Senior (n = 15)		
	Mean	±	SD	Mean	±	SD
Age	16.7	±	0.50	24.8	±	4.71
Height (cm)	167	±	4.90	169	±	5.31
Weight (kg)	62.4	±	7.29	64.9	±	7.87
BMI (kg/m^2^)	22.5	±	2.28	22.8	±	2.75

SD = Standard deviation; BMI: Body Mass Index.

**Table 2:** Descriptive statistics for the KIDMED, performance tests, and bone mineral density.

Study Variables	Total (N = 33)						ANCOVA Comparison (Adjusting for BMI)			
	Junior (n = 18)			Senior (n = 15)			Mean Difference	SE	t	*p* Value
Performance Test	Mean ± SD			Mean ± SD						
Yo-Yo (m)	382	±	81.4	414	±	109	−36.4	31.2	−1.17	0.253
Abalakov jump (cm)	28.5	±	5.69	35.1	±	6.89	−7.03	1.84	−3.83	<0.001
Handgrip (kg)	31.8	±	3.37	35.1	±	3.84	−3.11	1.20	−2.59	0.015
**Bone Quality (Ultrasounds)**										
BUA (dB/MHz)	137	±	9.55	139	±	11.7	−2.04	3.47	−0.601	0.552
SOS (m/s)	1666	±	37.2	1664	±	42.6	2.44	13.9	0.176	0.861
Stiffness (A.U)	139	±	13.7	138	±	17.9	−0.72	5.31	−0.135	0.894
**Mediterranean Diet Adherence**										
KIDMED	7.33	±	1.61	6.27	±	2.05	1.14	0.601	1.89	0.068

SD = Standard deviation; Yo-Yo IR: intermittent recovery test; KIDMED: Mediterranean Diet Quality Index; BUA: Broadband ultrasound attenuation; SOS: Speed of sound; BMI: Body Mass Index; N = total number of the sample; n = number of players on each team; t = t student; mean differences were significant at *p* < 0.05.

**Table 3:** Position-related differences in variables.

	Goalkeepers (n = 6)			Wings (n = 10)			Specialist (n = 8)			Pivots (n = 6)			Defenders (n = 3)			ANCOVA (Adjusted by BMI)		
Study Variables	Mean	±	SD	Mean	±	SD	Mean	±	SD	Mean	±	SD	Mean	±	SD	F	*p*	ηp2
**Body Composition**																		
BMI (kg/m^2^)	24.9	±	3.60	22.2	±	2.50	22.3	±	1.45	21.8	±	1.72	21.8	±	1.31			
**Performance Test**																		
Yo-Yo (m)	313	±	46.8	412	±	103	405	±	104	400	±	63.2	480	±	80.0	1.18	0.343	0.153
Abalakov jump (cm)	30.9	±	6.06	30.7	±	8.73	29.6	±	4.73	34.6	±	7.73	34.1	±	8.34	0.821	0.523	0.108
Handgrip (kg)	35.3	±	5.85	31.6	±	3.48	33.6	±	3.21	32.8	±	3.40	35.2	±	2.28	0.751	0.566	0.100
**Bone Quality (Ultrasounds)**																		
BUA (dB/MHz)	142	±	10.6	141	±	9.47	139	±	9.84	134	±	8.20	128	±	17.1	0.969	0.441	0.126
SOS (m/s)	1660	±	36.1	1673	±	38.9	1666	±	32.1	1659	±	43.7	1657	±	74.1	0.317	0.864	0.045
Stiffness (A.U)	138	±	17.1	143	±	15.1	139	±	11.6	133	±	12.6	129	±	32.2	0.522	0.720	0.072
**Mediterranean Diet Adherence**																		
KIDMED	7.67	±	1.63	6.80	±	2.20	6.25	±	1.83	6.67	±	1.75	7.33	±	2.08	0.273	0.893	0.039

SD = Standard deviation; BMI: Body Mass Index; Yo-Yo: intermittent recovery test; KIDMED: Mediterranean Diet Quality Index; BUA: Broadband ultrasound attenuation; SOS: Speed of sound; n = number of players of each position; np2 = partial eta (effect size); mean differences were significant at *p* < 0.05.

**Table 4:** Female beach handball players’ correlations.

		SENIOR								
		Weight	BMI	Yo-Yo	Abalakov	Handgrip	BUA	SOS	Stiffness	KIDMED
JUNIOR	Weight	n/a	0.868 *	−0.383	−0.493	0.320	0.333	−0.024	0.111	0.605 *
	BMI	0.856 **	n/a	−0.439	−0.512 *	0.359	0.661 *	0.343	0.316	0.524 *
	Yo-Yo	−0.359	−0.386	n/a	0.232	−0.101	−0.216	0.247	0.074	0.068
	Abalakov	−0.471 *	−0.630 *	0.653 *	n/a	0.133	−0.134	−0.011	−0.069	−0.477 *
	Handgrip	0.338	0.314	−0.089	−0.340	n/a	0.108	0.062	0.093	0.044
	BUA	0.242	0.057	−0.045	−0.196	−0.059	n/a	0.464 *	0.777 **	−0.079
	SOS	−0.046	0.029	0.602 *	0.011	−0.035	0.298	n/a	0.915 **	0.001
	Stiffness	0.078	0.049	0.438	−0.083	−0.054	0.681 *	0.894 **	n/a	−0.035
	KIDMED	0.051	0.186	0.174	−0.085	0.059	−0.335	0.057	−0.113	n/a

BUA: Broadband ultrasound attenuation; SOS: Speed of sound; BMI: Body Mass Index; Yo-Yo: intermittent recovery test; KIDMED: Mediterranean Diet Quality Index; n/a: not applicable; * = mean differences were significant at *p* < 0.05; ** mean differences were significant at *p* < 0.01. Gray color: numerical representation of the senior category. White color: numerical representation of the junior category.

## Data Availability

The data presented in this study is available on request from the corresponding author. The data are not publicly available due to is personal health information.
